# Dysregulated ΔNp63α Inhibits Expression of Ink4a/arf, Blocks Senescence, and Promotes Malignant Conversion of Keratinocytes

**DOI:** 10.1371/journal.pone.0021877

**Published:** 2011-07-15

**Authors:** Linan Ha, Roshini M. Ponnamperuma, Steven Jay, M. Stacey Ricci, Wendy C. Weinberg

**Affiliations:** 1 Division of Monoclonal Antibodies, Center for Drug Evaluation and Research/Food and Drug Administration (CDER/FDA), Bethesda, Maryland, United States of America; 2 SAIC-Frederick, Inc., Frederick, Maryland, United States of America; Roswell Park Cancer Institute, United States of America

## Abstract

*p63* is critical for squamous epithelial development, and elevated levels of the ΔNp63α isoform are seen in squamous cell cancers of various organ sites. However, significant controversy exists regarding the role of p63 isoforms as oncoproteins or tumor suppressors. Here, lentiviruses were developed to drive long-term overexpression of ΔNp63α in primary keratinocytes. Elevated levels of ΔNp63α *in vitro* promote long-term survival and block both replicative and oncogene-induced senescence in primary keratinocytes, as evidenced by the expression of SA-β-gal and the presence of nuclear foci of heterochromatin protein 1γ. The contribution of ΔNp63α to cancer development was assessed using an *in vivo* grafting model of experimental skin tumorigenesis that allows distinction between benign and malignant tumors. Grafted lenti-ΔNp63α keratinocytes do not form tumors, whereas lenti-GFP/v-ras^Ha^ keratinocytes develop well-differentiated papillomas. Lenti-ΔNp63α/v-ras^Ha^ keratinocytes form undifferentiated carcinomas. The average volume of lenti-ΔNp63α/v-ras^Ha^ tumors was significantly higher than those in the lenti-GFP/v-ras^Ha^ group, consistent with increased BrdU incorporation detected by immunohistochemistry. The block in oncogene-induced senescence corresponds to sustained levels of E2F1 and phosphorylated AKT, and is associated with loss of induction of p16^ink4a^/p19^arf^. The relevance of p16^ink4a^/p19^arf^ loss was demonstrated in grafting studies of p19^arf^-null keratinocytes, which develop malignant carcinomas in the presence of v-ras^Ha^ similar to those arising in wildtype keratinocytes that express lenti-ΔNp63α and v-ras^Ha^. Our findings establish that ΔNp63α has oncogenic activity and its overexpression in human squamous cell carcinomas contributes to the malignant phenotype, and implicate its ability to regulate p16^ink4a^/p19^arf^ in the process.

## Introduction

p63 is a p53 homologue. The *p63* gene contains the three functional domains homologous to those of p53, which mediate transactivation (TA), DNA binding (DBD) and oligomerization (OD) [1]. However, in contrast to p53's well established role as a tumor suppressor [2], p63 has been primarily considered a critical developmental regulator of epithelium. It is well understood that temporal regulation of individual p63 isoforms is required for both normal development and maintenance of mature epidermis. This is evidenced by studies in p63 null mice, which are born with severe abnormalities, including the lack of epidermis and many ectodermal derivatives, truncated limbs and craniofacial malformations [3], [4] and further supported by studies of postnatal keratinocytes in which p63 isoforms have been manipulated [5], [6].

Despite similarities in their structures, p63 is also distinct from p53 in its role in tumorigenesis. While *p53* is one of the most commonly mutated genes recognized to date in human malignancies, *p63* is rarely mutated in human cancers [7], though *p63* gene amplification and/or overexpression has been reported in human squamous cell carcinomas (SCC) of the head and neck, lung, cervix and skin [1], [8]–[12].

p63 is further distinct from p53 in its role in cell senescence. It is now well appreciated that senescence represents a potent anticancer mechanism to prevent tumor progression from premalignant to malignant lesions [13], [14]. In contrast to p53's established role in promoting this tumor-suppressive machinery, it has been shown that p63 deficiency leads to the activation of cell senescence and accelerated aging in mice [15].

Significant controversy exists regarding the role of *p63* as an oncogene or as a tumor suppressor gene [7]. In a study by Flores *et al.* [16], *p63^+/−^* mice were found to have increased susceptibility to spontaneous tumorigenesis. A complex tumor phenotype was observed in the mutant mice, which included squamous cell carcinomas, histiocytic sarcomas and adenomas. Mice heterozygous for null mutations in both *p53* and *p63* developed higher tumor burdens and had higher rates of metastases compared to *p63^+/+^/p53^+/−^* mice. These findings indicate that loss of p63 may cooperate in tumor formation with p53 loss-of-function. In contrast, an independent study by Keyes *et al.* [17] reported that p63^+/−^ mice were less prone to either spontaneous or chemically induced tumors. The neoplasms that did develop in *p63+/−* mice included lymphomas, sarcomas and carcinomas. In the latter study, mice heterozygous for null mutations in both *p63* and *p53* were found to have fewer tumors than *p63^+/+^/p53^+/−^* mice. These findings suggest that loss of p63 may prevent tumor formation mediated by p53 loss-of-function.

The complexity of *p63* contributes to the confusion surrounding the role of p63 in tumorigenesis [7]. p63 protein may refer to multiple variants arising from alternate promoter usage and/or alternative splicing. The *p63* gene is transcribed into two subclasses, TA and ΔN, which differ at the amino-terminus [1]. Additionally, alternative splicing gives rise to COOH-terminal variants p63α, -β and -γ within both TA- and ΔN-subclasses. TAp63 isoforms contain a p53-like N-terminal transactivation (TA) domain and are capable of transactivating known p53-responsive genes. ΔNp63 isoforms are transcribed from an alternate promoter and lack this transactivation domain, while still retaining transactivation activity [1], [18]–[20]. ΔN isoforms have also been shown to be capable of acting in a dominant-negative manner to block transactivation mediated by TAp63 isoforms as well as by p53 [1].

Accumulating evidence implies that the TA and ΔN isoforms have distinct or even opposing functions in neoplasia. Although it has been suggested that the tumor suppressor phenotype of p63 might come from TAp63 but not ΔNp63 isoforms [18], significant controversy still exists regarding the role of individual p63 isoforms in tumorigenesis. Decreased TAp63 levels have been linked to poor clinical outcomes in buccal and laryngeal squamous cell carcinomas [21], [22]. TAp63 functions as a robust mediator of cell senescence and inhibits tumorigenesis *in vivo* [23]. TAp63 isoforms have been found to promote cell apoptosis through death receptors and activating proapoptotic Bcl-2 family members [24]. A role of TAp63 in DNA damage-induced cell cycle arrest and cell death has also been demonstrated [25]. Conversely, Koster *et al.* reported, using an inducible transgenic mouse model, that embryonic induction of TAp63α causes keratinocyte hyperproliferation, and inhibits terminal differentiation [26], and that post-natal induction of TAp63α accelerates tumor development and progression [27].

ΔNp63 has been reported to be over-expressed in several different human cancers [8], [12]. ΔNp63α is the predominant isoform in basal keratinocytes, and its expression correlates with proliferation in human cancers [28]. ΔNp63α has been shown to promote survival in squamous epithelial malignancy by repressing a p73-dependent proapoptotic transcriptional program [29]. However, it has also been reported that ΔNp63α promotes basal keratinocyte withdrawal from the cell cycle and commitment to terminal differentiation or apoptosis [30], [31].

We have previously reported that transient overexpression of ΔNp63α causes enhanced cell proliferation and inhibition of morphological and biochemical differentiation in primary mouse keratinocytes [20], [32]. The long-term biological effects of ΔNp63α and the consequences of it's overexpression, as seen in human squamous cell carcinomas, remain unknown. In this study we apply a well-established model of squamous cancer to elucidate the role of elevated levels of p63 in distinct stages of tumorigenesis. We report that sustained elevated levels of ΔNp63α in normal cells block the p16^ink4a^/p19^arf^ pathways and promote keratinocyte survival. However, elevated levels of ΔNp63α alone are insufficient to confer a tumor phenotype *in vivo*. In addition to its effect on replicative senescence, elevated ΔNp63α blocks oncogene-induced senescence and associated induction of p16^ink4a^/p19^arf^, and cooperates with v-ras^Ha^ to enhance malignant conversion *in vivo*. These findings indicate an oncogenic role for the elevated levels of ΔNp63α that have been observed in human squamous cell cancers.

## Results

### Lentivirus infection drives long term and stable gene expression in primary keratinocytes

**Figure 1:**
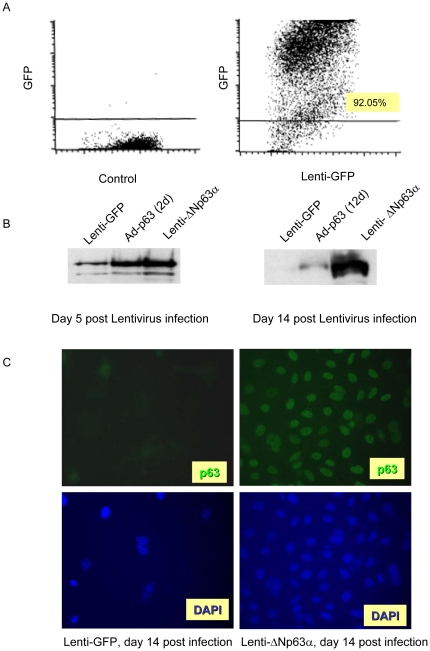
Lentivirus infection drives long term and stable gene expression in primary keratinocytes. A, Primary mouse keratinocytes were infected with lentivirus encoding GFP, and infection efficiency was assessed by FACS analysis at day 5 post-infection. Y-axis indicates the intensity of GFP, X-axis represents forward angle light scatter. B, Primary keratinocytes were infected with lentivirus encoding GFP or ΔNp63α and whole cell protein was collected at day 5 and 14 and analyzed for p63 expression by western blot. C, Primary keratinocyte cultures expressing lenti-GFP or lenti-ΔNp63α were fixed at 14 days post-lentiviral transduction and incubated with anti-p63 antibody, followed by a secondary antibody conjugated with Alexa-488. The cells were then stained with DAPI to visualize the cell nuclei.

To mimic the sustained high level expression of ΔNp63 that has been observed in human SCC, lentiviruses encoding green fluorescent protein (lenti-GFP) or ΔNp63α (lenti-ΔNp63α) were developed and their ability to drive long-term gene expression was assessed in cultures of primary epidermal keratinocytes. Under optimized conditions, the infection efficiency as assessed with lenti-GFP was 92.05% in primary keratinocytes five days following lentiviral infection (Figure 1A). Lenti-ΔNp63α expression was monitored over time by western blotting (Figure 1B). At five days following infection, lenti-ΔNp63α levels resembled those at the peak of adenoviral-driven gene expression (days 2–3, left panel), and remained stable for at least 14 days (right panel). Consistent with the FACS analysis shown for lenti-GFP in figure 1A, lenti-ΔNp63α expression observed by immunohistochemistry was distributed uniformly across the cell population (Figure 1C).

### Long-term ΔNp63α overexpression promotes survival and blocks replicative senescence of primary keratinocytes

**Figure 2:**
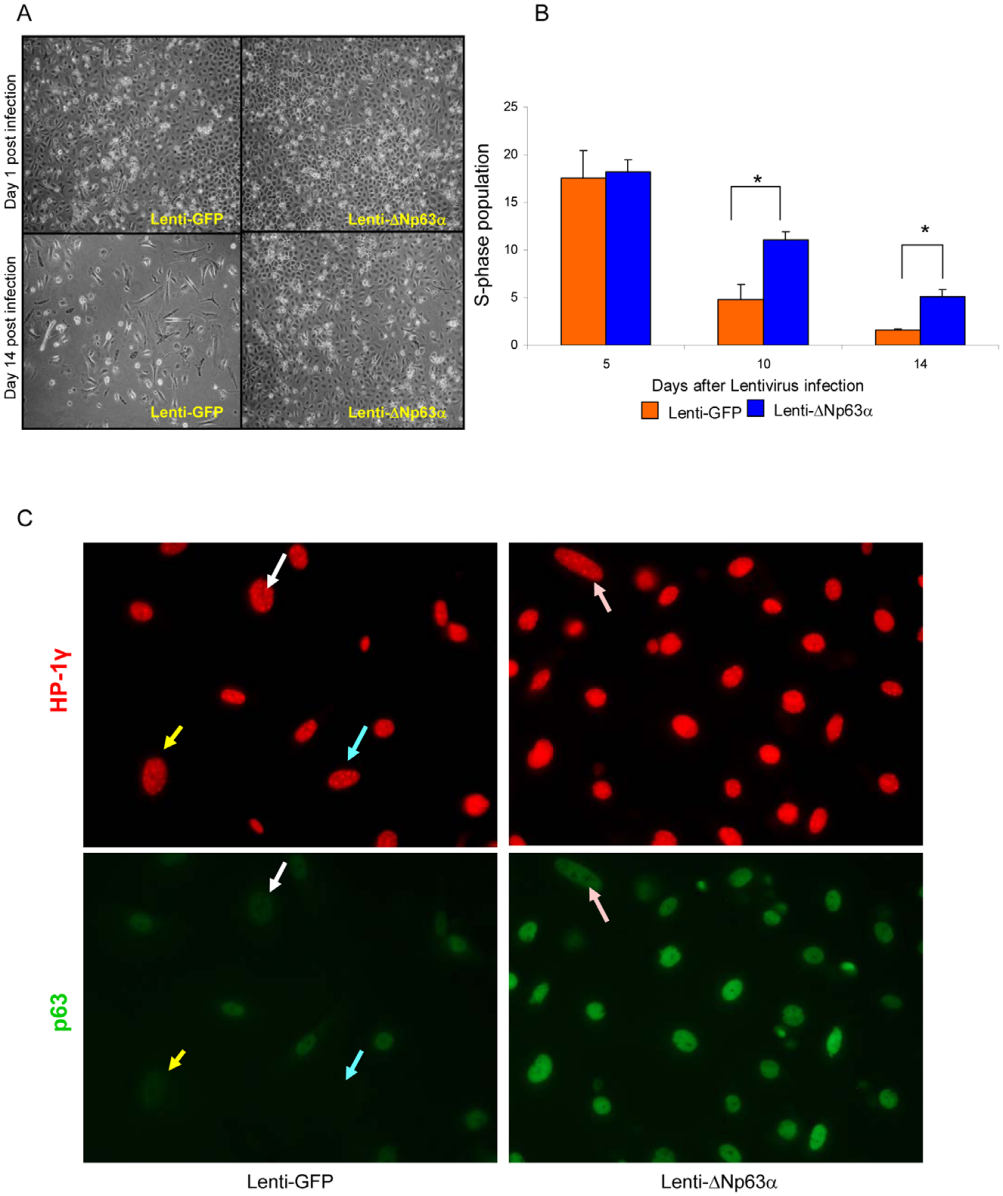
Dysregulated expression of ΔNp63α promotes survival and blocks replicative senescence of primary keratinocytes. A, Phase morphology of lenti-GFP- and lenti-ΔNp63α expressing keratinocytes cultured for 1 and 14 days post-infection. Results shown are representative of three independent experiments. B, Quantification of BrdU positive cells in lenti-GFP or lenti-ΔNp63α cultures at timepoints noted following lentiviral gene transduction. Data shown represent the means ± S.E. of three independent experiments. * indicates a statistically significant difference between lenti-GFP and lenti-ΔNp63α infected cells at *p*<0.05. C, Primary keratinocytes expressing lenti-GFP (left panel) or lenti-ΔNp63α (right panel) were fixed at 14 days post-lentiviral transduction. The cell senescence status was assessed by the presence of HP-1γ nuclear foci (red). The cells were double stained with p63 antibodies (green). Matched arrows indicate the same cell stained with both different antibodies. The image presented is representative of three independent experiments.

The effect of elevated levels of ΔNp63α on long-term *in vitro* survival of primary keratinocyte cultures was assessed by microscopic evaluation over 14 days following introduction of lenti-GFP or lenti-ΔNp63α. As shown in Figure 2A, lenti-ΔNp63α cultures remain confluent and appeared healthier at 14 days compared to lenti-GFP control cultures. Proliferation status was assessed by BrdU uptake followed by FACS analysis (Figure 2B). The S-phase population was initially similar across the two cell populations, as seen 5 days following introduction of the lentiviruses (18.18% *vs.* 17.58% in lenti-ΔNp63α- *vs.* lenti-GFP-expressing keratinocytes, respectively). While the S-phase population declined in both cell populations over 2 weeks in culture, it was maintained at higher levels in lenti-ΔNp63α cultures *vs.* the parallel lenti-GFP control cultures (11.12% *vs.* 4.12% BrdU-positive cells, respectively, at 10 days; 5.12% *vs.* 1.59% BrdU-positive cells, respectively, at day 14).

The continually decreasing BrdU uptake levels in both lenti-ΔNp63α and control cultures indicate that other mechanisms, in addition to cell proliferation, are involved in the prolonged maintenance of ΔNp63α overexpressing cells. Therefore, we tested the notion that overexpression of ΔNp63α facilitates the capacity of the cell to overcome replicative senescence, as originally described by Hayflick in cultured cells [33]. A previous study by Keyes *et al.*, showed that inactivation of all p63 isoforms in mice lead to cellular senescence and accelerated aging [15]. However, the role of individual p63 isoforms in cellular senescence has remained unclear. Here, keratinocytes were infected with lenti-GFP or lenti-ΔNp63α, and the cell senescence status at day 14 was determined by the nuclear presence of prominent HP1γ-positive senescence-associated heterochromatin foci [34]. As shown in Figure 2C, distinct HP-1γ nuclear foci were observed in control keratinocytes that express lenti-GFP, but not in keratinocytes that express lenti-ΔNp63α. Double staining with both HP-1γ and p63 antibodies demonstrated that the formation of HP-1γ foci is associated with lower p63 expression (Figure 2C, arrows).

**Figure 3:**
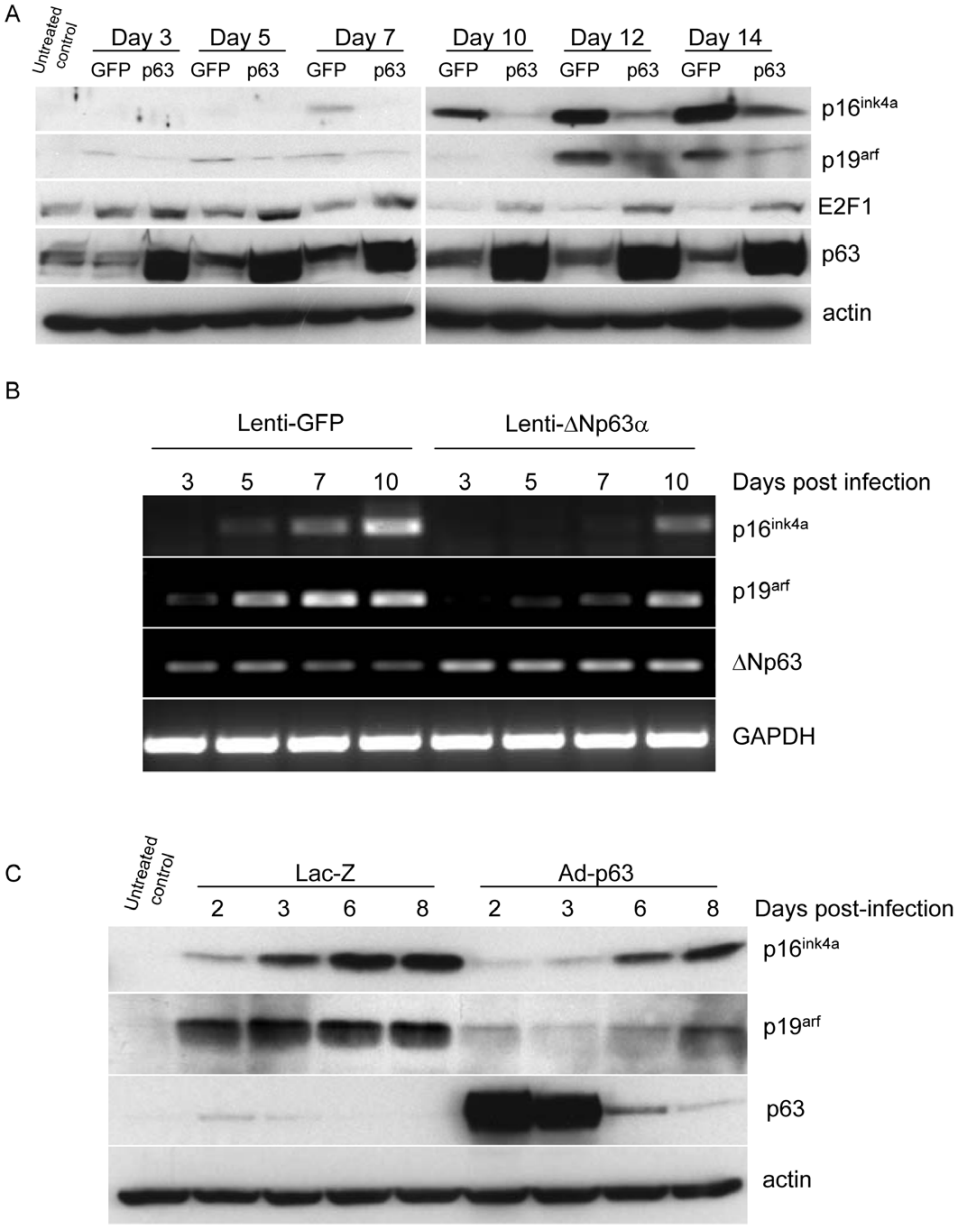
Overexpression of ΔNp63α inhibits the up-regulation of p16^ink4a^ and p19^arf^ associated with cell senescence. A, Whole cell protein was collected from primary keratinocytes expressing lenti-GFP or lenti-ΔNp63α at timepoints indicated following lentiviral gene transduction. Expression levels of p16^ink4a^, p19^arf^, E2F1 and p63 were detected by western blot. Equal protein loading was confirmed by immunoblotting for β-actin. B, Primary keratinocytes were infected with lentivirus encoding ΔNp63α or GFP at day 3 after plating. Total RNA was harvested at day 3, 5, 7 and 10 post-infection and reverse transcribed. Expression of p16^ink4a^ and p19^arf^ was determined by PCR amplification. PCR amplification of GAPDH was used as a loading control. C, Primary keratinocytes were cultured for 7 days after plating and then infected with adenovirus encoding ΔNp63α or LacZ. Whole cell lysates were collected at day 2, 3, 6 and 8 post-adenovirus infection (equivalent to day 9, 10, 13 and 15 post-plating). The expression levels of p16^ink4a^, p19^arf^ and p63 were detected by western blot. Equivalent protein loading was confirmed by immunoblotting for actin.

Senescence in most cells is regulated through some combination of activities within the RB and p53 pathways, but frequently operates in a context-dependent and complex manner [13]. We examined the status of the RB and p53 pathways in keratinocytes that differ in their senescence status due to expression of either lenti-GFP or lenti-ΔNp63α. The induction of p53, p21, p16^ink4a^, p19^arf^ and E2F1 were examined by western blot. No induction of p53 or p21 was observed during the timepoints studied (data not shown). In contrast, up-regulation of p19^arf^ followed by p16^ink4a^ was observed beginning by day 3 in control lenti-GFP keratinocytes cultures (Figure 3A). This expression of p16^ink4a^ and p19^arf^ increased over time in lenti-GFP cultures, but was attenuated and delayed in lenti-ΔNp63α-expressing keratinocytes (Figure 3A). As expected, the upregulation of p16^ink4a^ and p19^arf^ correlates with decreased levels of E2F1. The down-regulation of E2F1 was delayed and attenuated in cells over-expressing ΔNp63α (Figure 3A). We further used RT-PCR to confirm that the regulation of p16^ink4a^ and p19^arf^ by ΔNp63α occurs at the transcriptional level. RT-PCR analysis on p16^ink4a^ and p19^arf^ transcription was carried out on primary keratinocytes at day 3, 5, 7 and 10 post-lenti-GFP or lenti-ΔNp63α infection. As shown in figure 3B, p16^ink4a^ and p19^arf^ mRNA began to increase in lenti-GFP cells at day 5 post-infection. This induction was delayed in lenti-ΔNp63α overexpressing cells. These findings are consistent with a previous report, using cells null for all p63 isoforms, demonstrating that p63 directly represses p16^Ink4a^ and p19^Arf^ expression [35]. To further challenge the relationship between ΔNp63α and p16^ink4a^/p19^arf^, a transient induction of ΔNp63α was achieved using adenoviral-mediated gene transduction and used to assess its impact on temporal expression of p16^ink4a^/p19^arf^. Primary keratinocytes were infected with adenoviral constructs encoding Lac-Z or ΔNp63α at day 7 after plating, when p16^ink4a^ and p19^arf^ levels normally begin to increase. The expression levels of p16^ink4a^, p19^arf^ and p63 were evaluated at days 2, 3, 6 and 8 after adenoviral infection (equivalent to days 9, 10, 13 and 15 after plating, and to the time point of day 6, 7, 10 and 12 following introduction of lentivirus in Figure 3A). As shown in Figure 3C, adenoviral-driven gene expression of ΔNp63α peaked at day 2 post-infection and then rapidly declined. In control Ad-lacZ cultures, both p16^ink4a^ and p19^arf^ levels were seen to increase 2 to 3 days following adenovirus infection, in association with the onset of senescence typically seen in these cultures 10 days after plating. This upregulation was abrogated in parallel cultures in which Ad-ΔNp63α had been introduced. As Ad-ΔNp63α levels declined, levels of both p16^ink4a^ and p19^arf^ were once again up-regulated.

### Elevated ΔNp63α levels enhance malignant conversion in v-ras^Ha^-expressing keratinocytes

**Figure 4:**
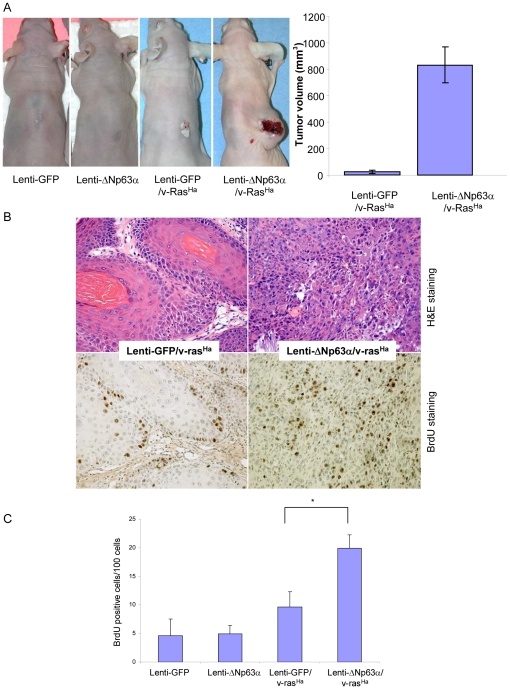
Elevated expression of ΔNp63α enhances malignant conversion in v-ras^Ha^-expressing keratinocytes. A, *In vivo* phenotype of ΔNp63α-overexpressing keratinocytes in the presence and absence of oncogenic ras. Primary mouse keratinocytes were infected with lentivirus encoding lenti-GFP or lenti-ΔNp63α or sequentially with retrovirus encoding v-ras^Ha^ followed by lenti-GFP or lenti-ΔNp63α. The final tumor phenotype was assessed at 5 weeks following grafting. Final tumor volumes at 4 weeks are presented as the mean tumor volume ± S.E. B, Upper panel, representative H&E sections of tumor tissues obtained from grafting sites. Lower panel, BrdU incorporation in grafted tissue samples. C, Quantification of BrdU incorporation levels. Data are presented as the mean % of BrdU positive cells ± S.E. * indicates a statistically significant difference between lenti-GFP/v-ras^Ha^ and lenti-ΔNp63α/v-ras^Ha^ groups at *p*<0.05.

The role of ΔNp63α in promoting cell proliferation and blocking the p16^ink4a^/p19^arf^ pathways of cell senescence, as shown in Figures 2 and 3, indicate that ΔNp63α might function as an oncogene in carcinogenesis. To determine the contribution of ΔNp63α to cancer pathogenesis, we applied a well established *in vivo* grafting model of experimental skin cancer that allows distinctions between benign and malignant tumor phenotypes. We introduced lenti-GFP or lenti-ΔNp63α into primary murine keratinocytes, either alone or in combination with retrovirus encoding a v-ras^Ha^ oncogene, and cultures were grafted onto the dorsal surface of nude mice in combination with cultured dermal fibroblasts as previously described [36]. No tumors were observed in grafts of lenti-GFP or lenti-ΔNp63α keratinocytes following grafting (Figure 4A and Table 1). Graft sites on 4 of the 15 animals that received lenti-GFP/v-ras^Ha^-expressing keratinocytes developed benign, well-differentiated papillomas with an average tumor volume of 25 mm^3^. In contrast, when v-ras^Ha^ was expressed in combination with lenti-ΔNp63α, all grafts (20 mice) gave rise to undifferentiated carcinomas with an average tumor volume of 831.9 mm^3^ (figure 4A, B and Table 1). The increased tumor size corresponded to increased BrdU incorporation, as detected by immunohistochemistry (Figure 4B and C), reflecting a higher proliferation status in these tumors.

**Table 1:** Elevated ΔNp63α levels enhance malignant conversion in v-ras^Ha^-expressing keratinocytes.

	Normal Skin	Papilloma	Carcinoma	Total	Conversion rate
Lenti-GFP	10	0	0	10	0%
Lenti-ΔNp63α	10	0	0	10	0%
Lenti-GFP/v-ras^Ha^	11 (73.3%)	4 (26.7%)	0	15	0%
Lenti-ΔNp63α/v-ras^Ha^	0	0	20 (100%)	20	100%

Tumor incidence and conversion rate of grafted keratinocytes are presented at 5 weeks post-grafting. Tumor phenotypes were confirmed histologically.

### Long term ΔNp63α overexpression blocks oncogene-induced senescence and supports long term survival of v-ras^Ha^-expressing primary keratinocytes

**Figure 5:**
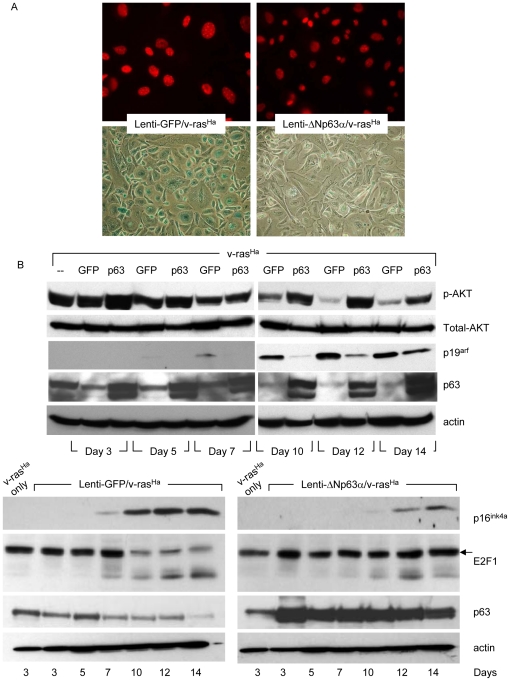
Long term ΔNp63α overexpression blocks oncogene-induced senescence. A, Primary keratinocytes were infected sequentially with retrovirus encoding oncogenic v-ras^Ha^, followed by lentivirus encoding GFP or ΔNp63α. Oncogene-induced senescence was assessed 14 days post-lentivirus infection by immunofluorescent analysis of nuclear foci of HP-1γ (upper panel), or enzymatic activity of SA-β-gal (lower panel). The images shown are representative of three independent experiments. B, Whole cell protein was collected from keratinocytes infected sequentially with retrovirus encoding oncogenic v-ras^Ha^ followed by lentivirus encoding GFP or ΔNp63α at timepoints indicated following lentiviral gene transduction. The levels of phosphorylated AKT, total AKT, p19^arf^ and p63 were detected by western blot (upper panels). p16^ink4a^ and E2F1 levels with corresponding p63 expression are presented in the lower panels. Equal protein loading was confirmed by immunoblotting for β-actin.

It has been well documented that normal keratinocytes undergo a transient hyperproliferation response followed by senescence after oncogenic v-ras^Ha^ activation [37]. Oncogene-induced senescence is now well appreciated to be a crucial barrier to malignant conversion [13]. The above results (Figure 4) demonstrate that elevated ΔNp63α levels enhance malignant conversion in v-ras^Ha^-expressing keratinocytes and suggest that ΔNp63α may play a role in blocking oncogene-induced senescence as well as replicative senescence. Primary keratinocytes were transduced with retrovirus encoding oncogenic v-ras^Ha^ followed by lentivirus encoding either lenti-GFP (Figure 5A, left) or lenti-ΔNp63α (Figure 5A, right). Oncogene-induced senescence was demonstrated at day 14 with the presence of nuclear foci of HP-1γ (Figure 5A, upper panels) or SA-β-Gal staining (Figure 5A, lower panels). While both of these well-defined markers of senescence were observed in lenti-GFP/v-ras^Ha^ cultures, they were diminished in lenti-ΔNp63α/v-ras^Ha^ keratinocytes.

p53 has been shown to direct cell fate between quiescence *vs.* senescence, and this has been linked to the status of the mTOR pathway [38], [39]. We evaluated levels of phospho-S6 as a marker of mTOR activity in v-ras^Ha^-expressing primary keratinocytes that had been transduced with either lenti-GFP or lenti-ΔNp63α. No change was observed in phospho-S6 levels due to the ΔNp63α status at days 5, 7 and 14 post-lentiviral infection, the timeframe during which control cultures are transitioning through senescence. These findings indicate that suppression of senescence in this system is not mediated *via* inhibition of mTOR (data not shown).

Similar to results seen with keratinocytes undergoing replicative senescence (Figure 3A), western blotting revealed up-regulation of both p16^ink4a^ and p19^arf^ in the presence of v-ras^Ha^ (lenti-GFP/v-ras^Ha^) that was attenuated and delayed in the additional presence of elevated ΔNp63α (Figure 5B). Furthermore, consistent with the increased proliferation status observed in tumors generated from lenti-ΔNp63α/v-ras^Ha^ keratinocytes (Figure 4B and C), the levels of E2F1 and phosphorylated AKT (serine 473) remain stable in lenti-ΔNp63α/v-ras^Ha^ keratinocytes, but start to decrease at day 7 in lenti-GFP/v-ras^Ha^ keratinocytes (Figure 5B).

**Figure 6:**
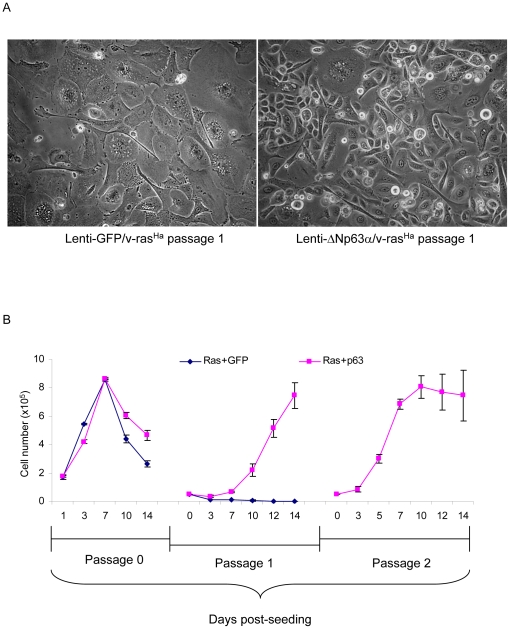
Overexpression of ΔNp63α supports long term survival of v-ras^Ha^-expressing primary keratinocytes. Primary keratinocytes were infected sequentially with retrovirus encoding oncogenic v-ras^Ha^ and lentivirus encoding GFP or ΔNp63α, as noted. On post-infection day 14, the cells were trypsinized and reseeded. A, cell morphology was recorded at day 3 after the initial reseeding. B, growth curves of keratinocytes expressing v-ras^Ha^ in conjunction with lenti-GFP or lenti-ΔNp63α. Cell numbers were counted at days 1, 3, 7, 10 and 14 post-lentivirus infection (passage 0), or at the same timepoints following each reseeding. Data are expressed as cell number per well and represent the means ± S.D. of two independent experiments.

We have shown that sustained dysregulation of ΔNp63α in keratinocytes supports cell proliferation and facilitates an escape from oncogene-induced senescence (Figure 5A and B). We tested whether co-expression of ΔNp63α and v-ras^Ha^ would cooperate to immortalize cells and allow multiple passaging of primary mouse keratinocytes. Primary cultures of v-ras^Ha^-expressing keratinocytes were transduced with lentiviruses encoding lenti-GFP or lenti-ΔNp63α. The cells were trypsinized 14 days following lentivirus introduction and reseeded. As shown in Figure 6A, lenti-GFP/v-ras^Ha^ keratinocytes display a senescence-like morphology 3 days after replating that is not observed in lenti-ΔNp63α/v-ras^Ha^ keratinocytes. Furthermore, lenti-ΔNp63α/v-ras^Ha^ keratinocytes re-attached and expanded for at least two passages, whereas lenti-GFP/v-ras^Ha^ keratinocytes stopped proliferation after the initial reseeding (Figure 6B).

### Loss of p19^arf^ cooperates with v-ras^Ha^ in malignant conversion, similar to lenti-ΔNp63α-expressing keratinocytes

**Figure 7:**
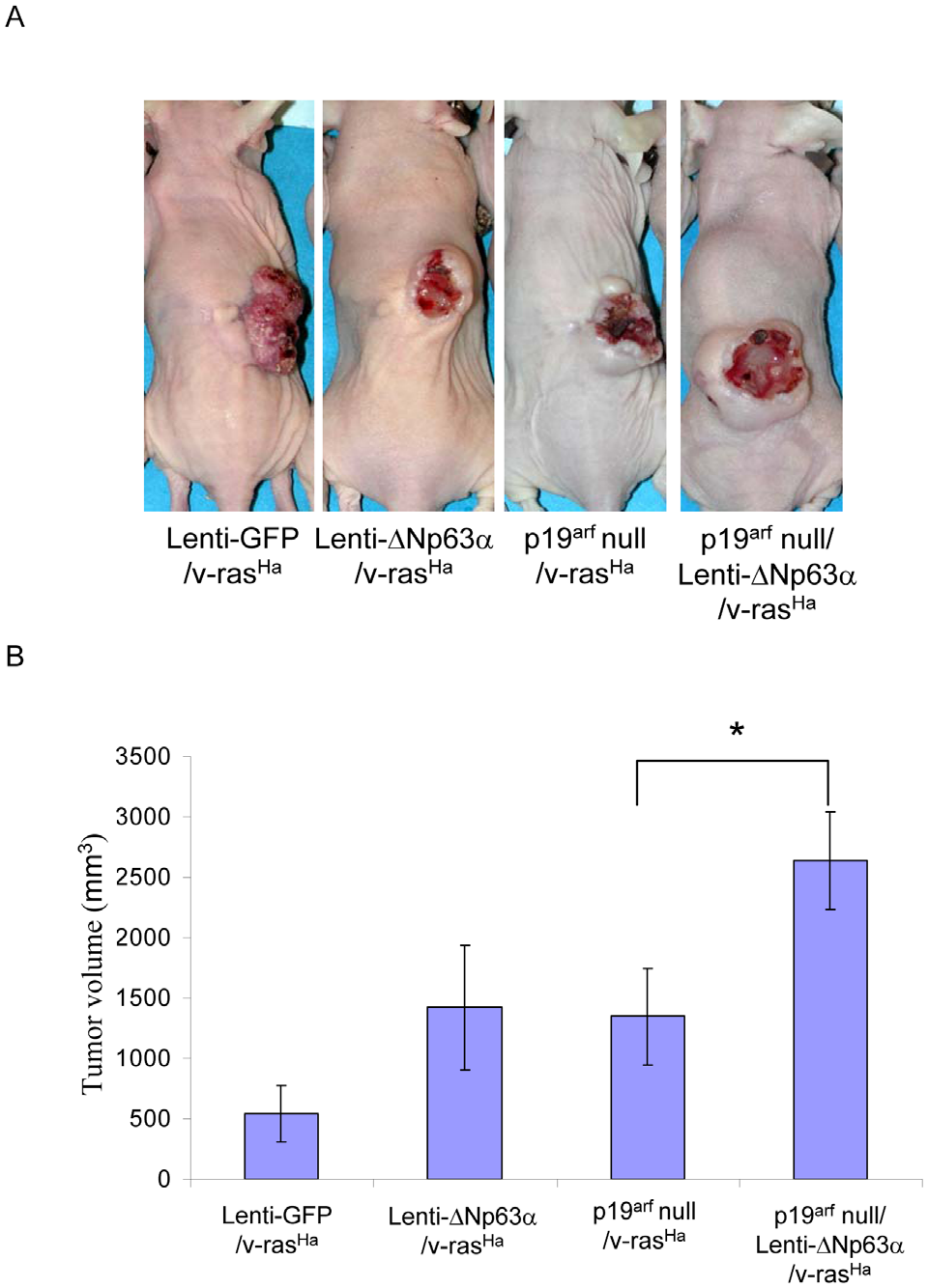
v-ras^Ha^-expressing p19^arf^ null keratinocytes display a tumor phenotype similar to v-ras^Ha^/ΔNp63α overexpressing keratinocytes. A, p19^arf^ null primary keratinocytes were infected with lenti-GFP or lenti-ΔNp63α in combination with v-ras^Ha^, and grafted onto the dorsal side of nude mice as previously described [36]. Wild type primary keratinocytes expressing lenti-GFP or lenti-ΔNp63α in combination with v-ras^Ha^ were used as negative and positive controls. The final tumor phenotype was assessed at 5 weeks after grafting. B, Final tumor volumes at 5 weeks are presented as the mean tumor volume ± S.E. * indicates a statistically significant difference between p19^arf^ null/v-ras^Ha^ and p19^arf^ null/lenti-ΔNp63α/v-ras^Ha^ groups at *p*<0.05.

To determine whether ΔNp63α-mediated downregulation of p16^ink4a^ or p19^arf^ could enhance malignant conversion, p19^arf^-null primary keratinocytes were transduced with v-ras^Ha^-encoding retrovirus, followed by lenti-GFP or lenti-ΔNp63α and grafted onto the dorsal surface of nude mice as previously described [36]. Wildtype primary keratinocytes expressing lenti-GFP or lenti-ΔNp63α in combination with v-ras^Ha^ were used as negative and positive controls. Out of 10 animals grafted with p19^arf^-null keratinocytes expressing lenti-GFP and v-ras^Ha^, 9 animals gave rise to undifferentiated carcinomas, which appeared similar to the tumor phenotype derived from keratinocytes overexpressing ΔNp63α. The remaining animal developed a well differentiated papilloma (Figure 7A and B, Table 2). A significantly larger tumor volume was observed in tumors derived from lenti-ΔNp63α/v-ras^Ha^-expressing p19^arf^-null keratinocytes when compared to tumors derived from p19^arf^-null cells that express v-ras^Ha^ alone (Figure 7B).

**Table 2:** Loss of p19^arf^ cooperates with v-ras^Ha^ in malignant conversion.

	Normal Skin	Papilloma	Carcinoma	Total	Conversion rate
Lenti-GFP/v-ras^Ha^	0	5	0	5	0%
Lenti-ΔNp63α/v-ras^Ha^	0	0	5	5	100%
p19^arf^ null/v-ras^Ha^	0	1	9	10	90%
p19^arf^ null/Lenti-ΔNp63α/v-ras^Ha^	0	0	10	10	100%

Tumor incidence and conversion rate of grafted p19^arf^ null keratinocytes are presented at 5 weeks post-grafting. Tumor phenotypes were confirmed histologically.

## Discussion

Expression of ΔNp63α is associated with proliferation of both normal and neoplastic epithelium, with elevated levels observed in human squamous cancer tissues [1], [8]–[12]. Here, we provide a mechanistic link between the elevated levels of ΔNp63α and development of squamous cell cancers. The grafting model applied in this study is relevant to the normal tissue distribution of p63, and allows the discrimination of genetic alterations that contribute to distinct stages of cancer development. We demonstrate that ΔNp63α overexpression alone does not confer a neoplastic phenotype upon normal primary keratinocytes under the conditions used, but enhances malignant conversion of benign tumors arising from primary murine keratinocytes that express oncogenic ras. This phenotypic change appears to be due, at least in part, to the ability of ΔNp63α to enhance cell survival through suppression of p16^ink4a^/p19^arf^, key mediators of cell senescence.

Cell senescence was initially described as a form of irreversible growth arrest in cultured human cells [33]. It is now understood that it can be prematurely induced by multiple stimuli including DNA damage, oxidative stress, and excessive mitogenic stimuli [40], [41], and represents a potent anticancer mechanism to prevent malignant conversion [14]. The complexities of the signaling pathways mediating the senescence response, and the dependence of this response on p53, are underscored by the contrasting results observed *in vitro* and *in vivo* in keratinocytes undergoing senescence induced by p63 ablation [15].

The inhibition of cell senescence by ΔNp63α is linked to its ability to repress the expression of p16^ink4a^ and p19^arf^ (Figure 5). While p16^ink4a^ and p19^arf^ play well-defined roles in controlling cell cycle and senescence, their regulation remains poorly understood. Our finding that ΔNp63α negatively regulates p16^ink4a^ and p19^arf^ in primary mouse keratinocytes is consistent with recent studies linking p63 to cellular senescence and organismal aging, in which keratinocytes of p63-deficient mice display increased levels of p16^ink4a^/p19^arf^ [15]. The ability of p63 to bind putative p53/p63 consensus sites on the p16^ink4a^/p19^arf^ promoters, as demonstrated by ChIP analysis using cells null for all isoforms of p63, raises the possibility that p63 acts as a direct repressor of the p16^ink4a^/p19^arf^ locus. A function for p16^ink4a^ downstream of p63 is also supported by the observation that germline disruption of both p16^ink4a^ and p19^arf^ was found to significantly alleviate the phenotypic consequences of p63 ablation during embryonic epidermal development [35]. Despite the above studies, the relationship between ΔNp63α and p16^ink4a^ in cancer remains unclear. Consistent with the findings reported here, a recent study by Keyes *et al.* [2] demonstrated that overexpression of ΔNp63α bypasses oncogenic ras-induced senescence and drives tumorigeneisis *in vivo*. However, in contrast to our results, Keyes *et al.* reported that the level of p16^ink4a^ remains stable in oncogenic ras and ΔNp63α expressing cells, and concluded that the inhibition of senescence by ΔNp63α occurs *via* an additional pathway, but not through modulating p16^ink4a^. This indicates the complexity of the pathways interacting with p63 family members, and underscores the need for additional studies to understand the role of p63 and its downstream effectors in tumorigenesis and senescence.

p63 is recognized as a regulator of epidermal cell fate and lineage commitment [42], [43]. The pathways involved in regulating epidermal development as well as homeostasis and differentiation of postnatal keratinocytes continue to be targets of active investigation [6], [28], [44]. Much effort has focused on the identification of downstream pathways of ΔNp63α contributing to neoplasia, including NFκB/c-Rel, EGF-R and COX-2 [45]–[47]. Furthermore, the ability of ΔNp63α to inhibit transcriptional regulatory activity of other p53 family members may contribute to a loss of genomic stability that could enhance progression to malignancy [1]. Our laboratory has previously demonstrated that NFκB/c-Rel mediates the aberrant growth regulation observed in ΔNp63α-overexpressing keratinocytes [43]. Here, we establish that the ability of ΔNp63α to overcome senescence and drive malignancy is linked to its downregulation of the p16^ink4a^/p19^arf^ pathways. As proof of principle that the loss of p16^ink4a^/p19^arf^ can contribute to ΔNp63α-induced malignant conversion, an *in vivo* grafting study showed that, similar to lenti-ΔNp63α-expressing keratinocytes, p19^arf^ null keratinocytes formed malignant carcinomas in the presence of v-ras^Ha^ (Figure 7). However, a significantly larger tumor volume was observed in tumors derived from lenti-ΔNp63α/v-ras^Ha^-expressing p19^arf^ null keratinocytes when compared to tumors derived from p19^arf^ null cells expressing v-ras^Ha^ alone (Figure 7B). Our results suggest that additional mechanisms, *e.g.* p16^ink4a^ or c-Rel, contribute to ΔNp63α -induced malignant tumor formation [45]. Future studies are required to dissect the relative roles of each individual downstream pathway influenced by p63 and their cooperative contribution to discrete steps of cancer pathogenesis.

## Materials and Methods

### Ethics Statement

All animal work was performed in accordance with NIH (National Institutes of Health) established guidelines, in accordance with accepted standards of humane animal care under protocols approved by the Animal Care and Use Committee of the Center for Biologics Evaluation and Research of the Food and Drug Administration.

### Cell Culture

Primary keratinocytes and dermal fibroblasts were isolated from the skin of 1–2 day-old C57B1/6NCr mice, cultured and plated as described [20]. Keratinocytes were maintained at a final concentration of 0.05 mM Ca^2+^. Fibroblasts were cultured for 9 days in DMEM prior to use in grafting studies.

Adenovirus encoding ΔNp63α has been previously described [20]. The replication-defective Psi-2 retroviral vector encoding v-ras^Ha^ was used to introduce a v-ras^Ha^ oncogene at day-2 post-plating as previously described [36]. The infection of adenovirus encoding Lac-Z or ΔNp63α was performed as previously described [20].

### Lentivirus construction and infection

Lentivirus encoding human ΔNp63α was generated by ligating the complete coding region of ΔNp63α cDNA excised from pBMNiGFP–human ΔNp63α plasmid into lentiviral expression vector 960-X5-685 (pSICO-FerH-eGFP). The constructed pSICO-hFerH-human ΔNp63α was verified *via* restriction enzyme mapping and sequencing analysis. A similarly constructed lentiviral vector encoding GFP was used as a matched control. Lentiviruses were produced in HEK293T cells (Open Biosystems, Huntsville, AL). The producer cells were washed and incubated with standard keratinocyte medium 24 hours before harvesting (SAIC, Inc., Frederick, MD).

Keratinocytes, 3 days post-plating, were incubated for 3 hours in lentivirus-containing supernatant and 4 µg/mL polybrene (500 µl/60 mm^2^ dish). Fresh medium was added at the end of the incubation. The induction of lenti-ΔNp63α was evaluated by western blot and immunofluorescent staining.

### *In vivo* grafting and tumor sample collection

Keratinocyte cultures were infected with retrovirus encoding v-ras^Ha^ one day prior to infection with lentivirus encoding either ΔNp63α or GFP. Cells were harvested six days following lentivirus infection and 4×10^6^ keratinocytes were mixed with 8×10^6^ cultured dermal cells and transferred to a grafting chamber freshly implanted onto the dorsum of a nude mouse [36], [48]. The chamber was removed 1 week following grafting. Tumor volume was measured every 3 days following the initial appearance of a tumor. Mice were injected with 250 µl of 50 mM 5-bromo-2′-deoxyuridine (BrdU, Sigma Chemical, St. Louis, MO) 1 hour prior to euthanasia and tumor harvest to allow immunohistochemical analysis of DNA synthesis in the grafted cells. Tumor tissues were collected 4 weeks after removing the chambers and fixed in formalin-buffered saline.

### BrdU incorporation

BrdU incorporation in the grafted tissue sample was detected by immunohistochemical staining using an antibody against BrdU (B44, BD Biosciences, San Jose, CA) and tissues were counter-stained with Contrast Green (KPL, Inc., Gaithersburg, MD). At least 100 basal cells were scored for each sample; in the case of undifferentiated carcinomas 100 cells/field were counted. BrdU incorporation *in vitro* was detected by Fluorescent Activated Cell Sorting (FACS) analysis as previously described [32].

### Senescence assays

For HP1γ detection, culture dishes were fixed with methanol at −20°C and incubated with antibody directed to HP1γ (MAB3450, Chemicon International, Billerica, MA), and p63 (4A4, Santa Cruz Biotechnology, Santa Cruz, CA). Cells were then incubated with a FITC- or rhodamine-labeled secondary antibody (Invitrogen) and viewed under a fluorescence microscope. Senescence-associated β-galactosidase (SA-β-gal) staining was performed as previously described [36].

### Keratinocyte growth curve

Keratinocytes were infected with retrovirus encoding v-ras^Ha^, followed by lenti-ΔNp63α or lenti-GFP. Cells were collected by trypsinization and counted using a hemocytometer (Hausser Scientific, Horsham, PA) at timepoints noted. At 14 days post-infection, the cultures were trypsinized and reseeded at 0.5×10^5^ cells per well, then counted as above at timepoints noted.

### Immunoblot Analysis

Immunoblot analysis was performed using standard procedures. The following primary antibodies were used: p63 (4A4, Santa Cruz Biotechnology), E2F1 (C-20, Santa Cruz Biotechnology), p16 (M-156, Santa Cruz Biotechnology), p19 (5-C3-1, Santa Cruz Biotechnology), β-actin (Clone AC-15, Sigma Chemical), phospho-AKT (ser473) (D9E, Cell Signaling Technology, Boston, MA), AKT (pan) (C67E7, Cell Signaling Technology) and phospho-S6 (Ser235/236) (D27.2.2E) and S6 (5G10) (Cell signaling Technology).

### RNA isolation and Reverse Transcription-PCR

Total RNA was harvested using TRIzol reagent (Invitrogen) and reverse transcribed (1 µg) using the SuperScript III first strand cDNA synthesis kit (Invitrogen) with an oligo(dT) primer. Expression of p16 and p19 mRNA was determined by PCR amplification using primers specific for the mouse p16/p19 gene (p16: sense 5′-GCTGCAGACAGACTGGCCA-3′; antisense 5′-GTCCTCGCAGTTCGAATCTG-3′) [49]. p19: sense 5′-GTCGCAGGTTCTTGGTCACT-3′; antisense 5′-ATGTTCACGAAAGCCAGAGC-3′) [50]. PCR amplification of GAPDH was used as a loading control. The PCR reaction program was as follows: denaturation, 94°C, 30 s; annealing, 53°C, 30 s; elongation, 72°C, 30 s for 35 cycles.
